# Neutralizing Dromedary-Derived Nanobodies Against BotI-Like Toxin From the Most Hazardous Scorpion Venom in the Middle East and North Africa Region

**DOI:** 10.3389/fimmu.2022.863012

**Published:** 2022-04-19

**Authors:** Rahma Ben Abderrazek, Ayoub Ksouri, Faten Idoudi, Sayda Dhaouadi, Emna Hamdi, Cécile Vincke, Azer Farah, Zakaria Benlasfar, Hafedh Majdoub, Mohamed El Ayeb, Serge Muyldermans, Balkiss Bouhaouala-Zahar

**Affiliations:** ^1^ Laboratoire des Biomolécules, Venins et Applications Théranostiques, Institut Pasteur Tunis, University Tunis El Manar, Tunis, Tunisia; ^2^ Laboratory of Cellular and Molecular Immunology, Vrije Universiteit Brussel, Brussels, Belgium; ^3^ Myeloid Cell Immunology Lab, Vlaams Interuniversitair Instituut voor Biotechnologie (VIB) Center for Inflammation Research, Brussels, Belgium; ^4^ Unité des Services Communs de Recherche (USCR) Séquenceur de Protéines, Faculté des Sciences de Sfax, Sfax, Tunisia; ^5^ Faculté de Médecine de Tunis, Université Tunis El Manar, Tunis, Tunisia

**Keywords:** *Buthus occitanus tunetanus*, scorpion toxin, VHH Dromedary library, phage display, nanobodies, neutralizing capacity

## Abstract

Scorpion envenoming is a severe health problem in many regions causing significant clinical toxic effects and fatalities. In the Middle East/North Africa (MENA) region, *Buthidae* scorpion stings are responsible for devastating toxic outcomes in human. The only available specific immunotherapeutic treatment is based on IgG fragments of animal origin. To overcome the limitations of classical immunotherapy, we have demonstrated the *in vivo* efficacy of NbF12-10 bispecific nanobody at preclinical level. Nanobodies were developed against BotI analogues belonging to a distinct structural and antigenic group of scorpion toxins, occurring in the MENA region. From *Buthus occitanus tunetanus* venom, BotI-like toxin was purified. The 41 N-terminal amino acid residues were sequenced, and the LD_50_ was estimated at 40 ng/mouse. The BotI-like toxin was used for dromedary immunization. An immune VHH library was constructed, and after screening, two nanobodies were selected with nanomolar and sub-nanomolar affinity and recognizing an overlapping epitope. NbBotI-01 was able to neutralize 50% of the lethal effect of 13 LD_50_ BotI-like toxins in mice when injected by i.c.v route, whereas NbBotI-17 neutralized 50% of the lethal effect of 7 LD_50_. Interestingly, NbBotI-01 completely reduced the lethal effect of the 2 LD_50_ of BotG50 when injected at 1:4 molar ratio excess. More interestingly, an equimolar mixture of NbBotI-01 with NbF12-10 neutralized completely the lethal effect of 7 and 5 LD_50_ of BotG50 or AahG50, at 1:4 and 1:2 molar ratio, respectively. Hence, NbBotI-01 and NbF12-10 display synergic effects, leading to a novel therapeutic candidate for treating *Buthus occitanus* scorpion stings in the MENA region.

## Introduction

Scorpion stings cause severe public health problems in many regions of the world, not only because of their high incidence in certain regions but also because of their potential to induce highly disturbing signs and symptoms, sometimes with fatal outcome especially in children (1–4). For example, 30,000 epidemiological cases are reported annually in Tunisia (27,000 for 2006, “Direction de soin et de santé de base, DSSB, Ministère de la santé”) of which 1,000 have systemic manifestations requiring admission to hospital (5). In Morocco, epidemiological data reported by the Poison Centre of Morocco reported 30,000–50,000 scorpionism cases annually, among which 1,000 cases were documented as severe envenomation (6). The severity of scorpion envenoming depends on scorpion species, venom composition, and the age of victims (7). Moreover, health services are barely able to provide an adequate remediation, as treatment of scorpionism is complex.

Approximately 1,500 scorpion species belonging to 18 families have been described. However, only 30 are considered dangerous to mankind, with 29 of them belonging to the family Buthidae (8–11). Only 11 account for fatal envenoming, including scorpions of the genus *Leiurus* in the Middle East (12), *Tityus* in South America (13, 14), *Centruroides* in North and Central America, *Mesobuthus* in Asia (15) (especially in India), *Parabuthus* in South Africa (3, 11), and *Androctonus* and *Buthus* in the Maghreb region of North Africa (16).

In the Maghreb region, the components of the venom are complex and “specific” for each scorpion species, those of the Buthidae family being the most toxic to humans. *Androctonus* and *Buthus* species are responsible for about 100,000 stings per year of which 1%–7% lead to death of the victim. The venom of this genus is very toxic, and associated symptoms of envenomation can include malignant hyperthermia, myocarditis, and pulmonary edema (17, 18). The venom of this family of Buthidae scorpions contains several low-molecular weight proteins (neurotoxins) that act mainly on two classes of ion channels: the sodium (Na+) and potassium (K+) voltage-gated channels (19–22). These channels conduct the electrical impulse in most excitable tissues, promoting permeability to ions, which initiates the action potential.

Based on their primary sequences, toxins from *Buthidae* have been classified in three distinct structural and antigenic groups: (i) group 1 comprising AahI, AahII, AahI, AahIII, and AahIV; (ii) group 2 with AahII and BotIII analogues; and (iii) group 3 represented by BotI, BotII, and BotXIV (23). The structural divergences that reflect the functional topographies of BotI-related toxins suggested a significant functional diversity and complexity of their structure–function relationships (24). Preventing stings is not possible because of the wide distribution of scorpions. Therefore, lifesaving approaches should focus on the treatment of the envenoming that occurs after the sting. A treatment depends on what is known about each venom contents because only a limited number of neurotoxins are responsible for its lethality. The current immunotherapeutic treatment of scorpion envenoming consists in administering purified polyclonal F(ab’)_2_ fractions prepared from equine hyperimmune sera. An important side effect of these antisera products is the early anaphylactic shock and late serum sickness (25). In addition, a lack of efficacy of the antiserum can occur in case of delay in transferring to health centers (26). To overcome these problems, investigators have attempted to replace the existing F(ab′)_2_ fragments by a better antibody format to combat scorpionism (27, 28). Successful therapy is aided by speedy administration of antivenom (29–31). The introduction of smaller, recombinant antigen-binding fragments derived from monoclonal antibodies is considered to be such an improvement on the current therapy. The VHH or nanobody is the smallest intact antigen-binding fragment derived from functional immunoglobulins (32, 33). Nanobodies have a small size, high affinity, and specificity for their cognate antigen, combined with a high sequence identity to human VH of family III (34), which make them a potential efficient agent in immunotherapy of scorpion stings (10, 25, 35–40). In this context, we have been interested in improving the efficacy of antivenoms by developing nanobodies specific to the most toxic molecules. To this end, nanobodies against the two n, AahI and AahII, purified from *Androctonus australis hector* scorpion venom (groups 1 and 2), were selected and characterized (35–38). Their neutralization and protection efficiency has been demonstrated. However, envenomation caused by *Buthus occitanus* scorpion is the most occurring sting in the MENA region (41). The current challenge is to develop nanobodies that specifically recognize and neutralize BotI-related toxins. This is essential to overcome the structural and antigenic diversity of the toxins within the venom, which may limit the antivenom efficacy. In this study, we first screened all the components extracted from *Buthus occitanus tunetanus* scorpion venom in order to identify the toxic molecules analogous to the BotI toxin. A novel BotI-like toxin that is able to interact with rabbit anti-BotI polyclonal antibodies has been further structurally investigated and used for dromedary immunization. The successful generation of two nanobodies against BotI-like protein is hereby reported and structurally modeled. Their expression yield and affinity have been estimated, and epitope mapping on the toxin has been determined by BIAcore measurements. Because *Buthidea* scorpions have overlapping territories and victims of scorpion sting have often no idea which scorpion caused the sting, further investigations were performed with BotG50 and AahG50 fractions. To this end, the best nanobody candidate with picomolar affinity and a strong neutralization activity was further used in equimolar mixture with the bispecific NbF12-10 to assess their neutralizing capacity against BotG50 and AahG50 lethal doses (37).

## Materials and Methods

### Animals

The pubertal male dromedary (*Camelus dromedarius*) was housed in Sousse, Tunisia. The Swiss mice, 8 weeks old, were provided by the “Institut Pasteur de Tunis”, Tunis, Tunisia. Immunization was conducted according to a well-established immunization protocol approved by the Biomedical Ethics Committee of the Institut Pasteur de Tunis and according to the 2010/63/EU Directive for animal experiments.

### Toxin Purification and Identification

The venom of the scorpion *Buthus occitanus tunetanus* was extracted with water and fractionated by Sephadex G-50 gel filtration. The majority of approximately 7-kDa molecular weight material was eluted in one fraction. This toxic fraction is referred to as BotG50. Protein concentration was estimated at 280 nm (1 OD_280nm_ corresponds to 0.54 mg/ml), as previously described (35). BotG50 was loaded on a Mono-S FPLC column and eluted using a linear gradient of ammonium acetate ranging from 0.05 to 0.5 M over a time span of 60 min. The toxicity of the major eluted fraction that is recognized by BotI-specific rabbit antibodies was assessed by intracerebroventricular (i.c.v) injection in Swiss mice and further purified on an high-performance liquid chromatography (HPLC) C8 column. A linear 15%–50% gradient of 0.1% trifluoroacetic acid (TFA) in acetonitrile run for 50 min was used. The N-terminal sequencing of purified toxin was performed by Edman degradation. The toxicity of the purified toxin was assessed by intracerebroventricular (i.c.v.) injection in Swiss mice.

### Camel Immunization

One 5-year-old male dromedary was used for immunization. An increasing amount of the BotI-like enriched toxin (from 200 to 400 µg) was administered by five subcutaneous (s.c.) injections at weekly intervals and then three times with 100 µg of highly purified toxin. Following the immunization program, a volume of 200 ml blood was collected from the jugular vein to prepare the blood lymphocytes, and serum was prepared as well.

### ELISA

The specificity of the immune sera (diluted from 1.500 to 1:20,000) was tested in an ELISA. Two micrograms of BotI-like enriched fraction and BSA (irrelevant antigen) were coated overnight at 4°C in wells of a Maxisorp plate. After the plates were washed five times with phosphate-buffered saline (PBS)–Tween 20 (0.05%) (PBS-T), the residual protein-binding sites in the wells were blocked by PBS, 5% milk. Then, 100 µl of serially diluted immune and non-immune sera was added to separate wells and incubated for 1 h at room temperature (RT). The wells were washed five times with PBS-T [PBS plus 0.1% (v/v) Tween-20] and incubated with an anti-LAMA phosphatase alkaline-conjugated antibody (Sigma, St. Louis, MO, USA) (1:8,000). The reaction was developed using TMB substrate (catalog number 555214, BD Biosciences, San Diego, CA, USA) for 15 min at room temperature and stopped with 100 µl/well of 2 N H_2_SO_4_. After incubation for 20 min at room temperature, titers were estimated by measuring the optical density at 450 nm with a plate reader (Thermo Electron Corporation Multiscan EX, MA, USA). All experiments were performed in duplicate, and the mean values and standard deviations were calculated.

### *Escherichia coli* Strains and Vectors

The *E. coli* strain TG1 was used to host the VHH library. The phage display vector pHEN4 was employed to construct the VHH library containing PelB leader signal to secrete the VHH in the periplasmic space, HA-tag for VHH-detection, and the M13 bacteriophage PIII gene downstream of an Amber stop codon (35, 37). The Nb genes selected by biopanning were recloned in the expression vector pHEN6 lacking the phage gene III and containing C-terminal His6-tag used for recombinant protein purification by immobilized-metal affinity chromatography (IMAC) (35, 37). The *E. coli* strain WK6 was used for the Nb genes expression.

### Library Construction, Panning, and Selection of BotI-Like-Specific Binders

The VHH library was cloned from peripheral blood lymphocytes according to previously described procedures (35, 37, 42–46). Briefly, 4 days after the last booster injection, a blood sample (200 ml) was taken in ethylene diamine tetraacetic acid (EDTA)-coated tubes, and the total peripheral blood mononuclear cells (PBMCs) were isolated with density gradient using Ficoll (catalog number P04-60500, Pan Biotech, Aidenbach, Germany). Subsequently, total RNA was extracted and purified. An amount of 50 μg of total RNA was reverse transcribed into cDNA with oligo-dT primer and the SuperScript II First-Strand Synthesis System for reverse transcription PCR (RT-PCR) (catalog number 18064-014, Invitrogen, Carlsbad, CA, USA). A two-step PCR amplification was performed to amplify VHH gene fragments. The first PCR was performed with a leader-specific primer, CALL001 (5′-GTCCTGGCTGCTCTT CTACAAGG-3′) and a CH2-specific primer, CALL002 (5′-GGTACGTGCTGTTGAACTGTTCC-3′). DNA fragments that corresponded to heavy chain antibody fragments (600 and 700 bp) were extracted from agarose gel (Qiaquick Gel Extraction kit, catalog number 28704, Qiagen, Hilden, Germany) and then used as template for second PCR with nested PCR primers: A6E (5′-GATGTGCAG CTGCAGGAGTCTGGRGGAGG-3′) and 38 (5′-GGACTAGTGCGGCCGCTGGAGACGGTGACCTGGGT-3′) specific for framework 1 and framework 4, containing *Pst*I and *Not*I recognition sites, respectively. The VHH genes were purified from agarose gel (Qiaquick Gel Purification kit, catalog number 28104, Qiagen, Hilden, Germany) and digested with *Pst*I and *Not*I restriction enzymes (catalog numbers R3140T and R3189M, New England Biolabs, Hitchin, UK, respectively). The digested VHH amplicons were cloned into similarly digested pHEN4 phagemid vector using a molar ratio 1:3 in favor of the PCR amplicon. The ligation product (T4 DNA ligase, catalog number 15224-041, Invitrogen, Carlsbad, CA, USA) was transformed into freshly prepared electrocompetent *E. coli* TG1 cells and plated on LB-agar supplemented with (2% w/v) glucose (catalog number G8270, Sigma-Aldrich, MO, USA) and 100 µg/ml ampicillin (catalog number 271896, Sigma-Aldrich, St Louis, MO, USA). The capacity of the constructed library as a reliable source of antibodies was estimated by counting the colony numbers on LB-ampicillin (100 g/ampicillin) agar plates. All colonies were scraped into LB-agar medium containing 20% glycerol and were stored at −80°C for further use.

The cloned VHH repertoire was expressed on phage after infecting an aliquot of the library with 10^12^ M13K07 helper phages (catalog number 170-3578, New England Biolabs, UK) and incubated 30 min without shaking followed by resuspending the cells in a fresh medium (containing 100 µg/ml of ampicillin and 70 µg/ml of kanamycin) and rapid shaking overnight at 37°C. Recombinant phages were prepared by polyethylene glycol (PEG)/NaCl precipitation. The virions were harvested, resuspended in 1 ml sterile PBS and titrated and used as input phage for the panning process. Phage virions carrying toxin-specific VHHs were enriched by four consecutive rounds of panning on 10 μg of toxin and immobilized in a well of a microtiter plate (catalog number M5785-1CS, Sigma-Aldrich, MO, USA). After each selection, the toxin-specific virions were eluted with 100 mM triethylamine (pH 10.0, catalog number T0886, Sigma-Aldrich, MO, USA) and immediately neutralized with 1.0 M Tris–HCl, pH 8.0 (catalog number CE234, Gene ON, Germany). Phage particles were finally used to infect exponentially growing *E. coli* TG1 cells. An aliquot was properly diluted and plated on LB agar with ampicillin. The enrichment of phage particles carrying an antigen-specific VHH was assessed by comparing the number of virions eluted from wells coated with BotI-like-toxin versus non-coated wells. Polyclonal phage ELISA using eluted phage from each round of panning (output phages) was carried out to follow the enrichment during the consecutive rounds of panning. The antigen-specific bound phages were detected with horseradish peroxidase (HRP)-conjugated anti-M13 monoclonal antibody (catalog number 27942101, Sigma-Aldrich, MO, USA). After the fourth round of biopanning, individual colonies were randomly chosen and cultured in Terrific Broth for a periplasmic extract ELISA in order to detect antigen-specific Nb-containing clones. The expression of soluble periplasmic Nbs was induced with 1 mM isopropyl-D-thiogalactopyranoside (IPTG, catalog number 2900245 5 PRIME, Germany). Solid-phase ELISA of each periplasmic extract was carried out on BotI-like toxin (1 μg/ml), using a mouse anti-HA antibody (catalog number H9658, Sigma-Aldrich, MO, USA) and goat anti-mouse IgG-peroxidase antibody (catalog number A9044, Sigma Aldrich, MO, USA).

### VHH Sequence Analysis and Structural Computational Study

The VHH sequences of clones that scored positive in periplasm extract ELISA were determined using the IPTomics platform of Institut Pasteur de Tunis facilities (ABI Prism 3100 genetic analyzer, Applied Biosystems, Foster City, CA, USA). The VHH nucleotide sequences were obtained using the ABI PRISM™ BigDye Terminator v3.1 Cycle Sequencing Reaction Kit (catalogue number 4337454, Applied Biosystems, USA). Pairwise sequence alignment was performed using needle emboss application to identify NbBot-01 and NbBot-17 similarities that could support structural and functional properties of highly specific binding to BotI toxin (47).

Nanobody structural models were designed using a specific advanced Rosetta antibody application (48) tool from ROSETTA 3.8” software package^1^. A super computer server with a high computing capacity was used to obtain high-resolution models. Based on system energy, the 3D model with best Rosetta scoring was selected as previously described (49). Structural shapes and crucial positions were visualized and using PyMOL software (50).

### Expression and Purification of Nanobodies

The VHH genes were sub-cloned into the pHEN6 (encoding a C-terminal His-tag) expression vector using PstI and BstEII (catalog number R0162M, New England Biolabs, UK) restriction enzymes. The plasmid constructs were transformed into *E. coli* WK6. Nb production was performed in shake flasks by growing each recombinant bacteria in Terrific Broth medium (TB, catalog number 743-29175, BD Biosciences, FL, USA) supplemented with ampicillin (100 μg/ml) and 0.1% glucose until the absorbance at 600 nm reached a value between 0.5 and 0.9. The Nb periplasmic expression was subsequently induced with 1 mM IPTG, O/N at 28°C. The bacteria were collected by centrifugation at 4,000×*g* for 15 min, and periplasmic protein was extracted by osmotic shock and loaded on a His-Select column (NiNTA, catalog number 1018544, Qiagen, Hilden, Germany). The His-tagged BotI-like toxin-specific Nbs were eluted with 500 mM imidazole (catalog number I-0125, Sigma Aldrich, MO, USA) following dialysis with PBS (12 kDa cutoff membrane) (catalog number D9527-100FT, Sigma Aldrich, MO, USA). The final yield of the purified Nb was determined from UV absorption at 280 nm using the theoretical extinction coefficient and molecular weight, as calculated from the amino acid content of the Nb clone.The purity and identity of the nanobody was checked by Western blotting.

### Affinity Measurements and Epitope Mapping

The binding kinetics between toxin and the two selected nanobodies were measured by SPR on a biosensor instrument (BIAcore T200). The BotI-like toxin antigen was immobilized on a CM5 sensor chip using *N*-hydroxysuccinimide-*N*-ethyl-*N*′-(dimethylaminopropyl)-carbodiimide chemistry until 70 resonance units were immobilized. The antibody fragments were diluted twofold in HEPES-buffered saline (HBS) buffer (10 mM HEPES pH 7.5, 150 mM NaCl, 3.5 mM EDTA, and 0.005% Tween-20) at concentrations between 0.49 and 125 nM (with 31.25 nM in duplicate to confirm reproducibility and two HBS buffer as negative control). Measurements for all nine different concentrations were performed at a flowrate of 30 µl/min in HBS buffer. The binding sensorgrams were used to calculate the kinetic rate constants, *k_on_* and *k_off_* (using the BIA-evaluation software version 4.1 from BIAcore), from which we calculated the equilibrium binding constant, *K_D_*. The sensorgrams obtained with concentrations of Nb that yielded the R_max_ value were omitted for the fittings. Fittings were with 1:1 stoichiometry and with a 1:1 stoichiometry with RI^2^ and drift. The latter gave consistently better chi^2^ values and was retained. After each measurement, the residual Nbs were cleared from the toxin on the surface with 50 mM NaOH (35, 37).

For epitope binning of Nbs against toxin, an excess of a first Nb was injected (100× K_D_ value). After the binding of this Nb reached equilibrium, a mixture of the first Nb with a second Nb was injected (35). If the two Nbs recognize a different epitope, the signal of the Nb mixture increases with the signal of the first Nb alone. No difference in the signal is observed in cases where the epitopes, recognized by the two Nbs overlap each other.

### Toxicity and Neutralization Assays of BotI*-*Like Toxin, BotG50, and AahG50 Fractions

Mouse experiments have been approved by the Ethics Committee of Pasteur Institut of Tunis (2019/12/I/LR16IPT). Toxicity of increasing amounts of purified toxin, BotG50, or AahG50 fractions were assessed by i.c.v. injection into Swiss mice, according to the 3R rules. The LD_50_ values were measured and corresponded to the amount of pure toxin, BotG50, or AahG50, whereby 50% of the mice survived.

For the neutralization assays, we mixed increasing lethal doses of purified toxin, with approximately 2 or 4 molar excess of selected Nb, and incubated the mixture for 1 h at 37°C before i.c.v. injection in cohorts of four mice. Furthermore, increasing lethal amounts of BotG50 and AahG50 were incubated with a 2 or 4 molar excess of an equimolar mixture of NbBotI-01 and NbF12-10 (37).

## Results

### Venom Purification

**Figure 1:**
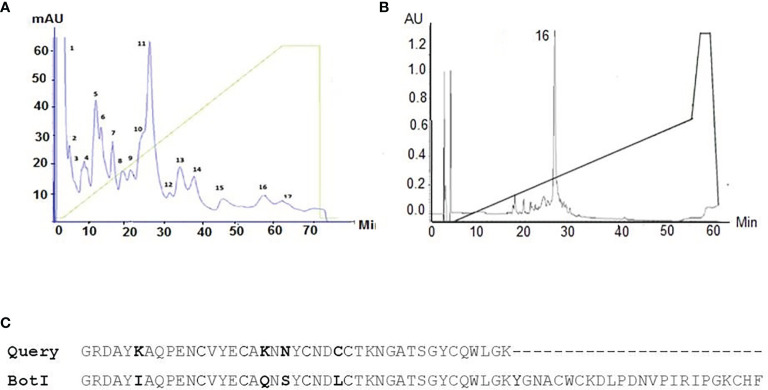
**(A)** Chromatographic profile obtained by fractionation of BotG50 on a Mono-S cation exchange column. **(B)** The major eluted fraction that is recognized by BotI-specific rabbit polyclonal antibodies was further purified on a reversed phase HPLC C8 column. Analytical C8 HPLC runs with the Peak16 eluted at 26 min and 29% of acetonitrile-TFA (0.1%), identified as BotI-like toxin. **(C)** Amino acid sequence of target molecule compared with BotI scorpion toxin sequence.

The toxic fraction of the *Buthus occitanus tunetanus* venom obtained from Sephadex G-50 (i.e. BotG50) was further fractionated on a Mono-S column (**Figure 1A**). The LD_50_ of the major eluted fraction F11 that is recognized by BotI-specific rabbit antibodies is 50 ng/mouse. This LD_50_ is the lowest from all tested fractions. Thus, this fraction was further injected into a C8 reversed phase column. The major symmetric peak 16-F11 is eluted at a retention time of 26 min and 29% of acetonitrile-TFA (0.1%) (**Figure 1B**).

The 41 amino acid residues at the N-terminal sequence of the purified toxin show a high identity to the primary sequence of the BotI toxin, exceeding 90% (**Figure 1C**). The LD_50_ of the BotI-like toxin is estimated at 40 ng/mouse.

### Titer of Immune Sera

The immune response of the dromedary immunized with BotI-like toxin was assessed using the serially diluted sera collected at the end of the immunization program. An antigen-specific response of high titer was elicited and estimated to be approximately 1/10,000 (**Supplementary Figure 1**).

### Library Construction and Selection of Specific BotI-Like Toxin Binders

VHH amplicons, from blood lymphocytes prepared from bleedings after the last immunization, were ligated into pHEN4 vector (**Supplementary Figures 2A, B**) and transformed in *E. coli* (35, 37, 51). A VHH library of 3.10^6^ individual transformants was obtained, and the percentage of the clones containing a vector with a VHH coding sequence was estimated to be approximately 87%. The results of the polyclonal phage ELISA from phages eluted after each round of biopanning confirmed the enrichment of antigen-specific Nb clones (**Supplementary Figure 3**). From 24 individual colonies, randomly picked after the third and the fourth round of panning, the periplasmic proteins were checked by ELISA for the presence of binders to the BotI-like toxin. All these extracts yield a strong signal in ELISA, which reached at least two times the background signal.

### Sequence Analysis of Selected Nanobodies

**Figure 2:**
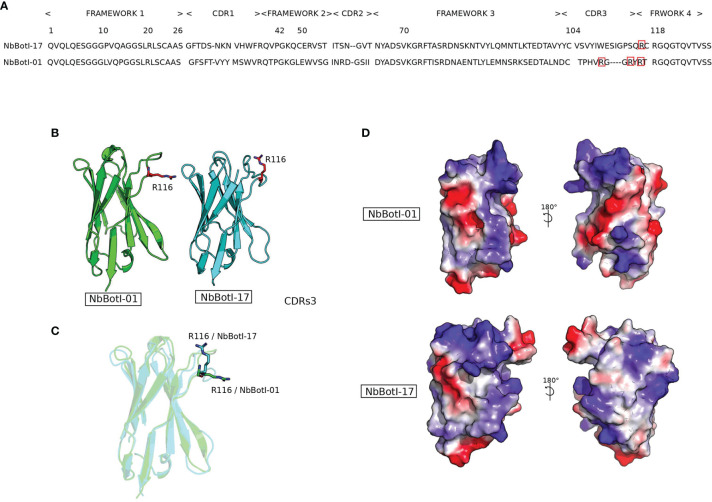
Representation of two nanobody 3D structure models NbBotI-01 and NbBotI-17. **(A)** Pairwise sequence alignment illustrates the identity and similarity of amino acids residues between NbBotI-01 and NbBotI-17 especially in the CDR3 regions. **(B, C)** Nanobody structures in the same orientation are presented in cartoon mode with positively charged Arg116 of CDR3 shown in stick representation. **(D)** Electrostatic potentials (red, negative charges; blue, positive charges) and hydrophobic coloring (gray) were applied on the surface area of nanobody structures and visualized using the PyMOL molecular graphics.

The nanobody sequences were aligned according to ImMunoGeneTics (IMGT) (52) and grouped in two distinct clusters according to their CDR3 sequence homology and referred to as NbBotI-01 and NbBotI-17 (**Figure 2A**). Based on the pairwise sequence alignment, the CDR sequences of these two nanobodies reflect an independent B-cell lineage origin. NbBotI-17 possessed all the VHH hallmark amino acids within its framework region-2 with a Cys at position 50 and an extra Cys at the end of the CDR3, besides the conserved Cys23 and Cys104. Remarkably, NbBotI-01 contains only VH hallmark amino acid in its framework region-2 (Val42, Gly49, Leu50, and Trp52) and a short CDR3 length of 11 amino acids, closely related to human VH CDR3 lengths. However, the presence of Arg118 instead of the conserved Trp118 (i.e., the VL anchoring site in VH) strongly suggested that NbBotI-01 will fail to associate with a VL domain and was therefore considered as a VH-like VHH domain (53, 54) (**Figure 2A**).

Interestingly, based on the 3D models, the NbBotI-01 CDR3 region displays the presence of three positively charged residues Arg109, Arg114, and Arg116 versus only one Arg116 for the NbBotI-17 CDR3 (**Figures 2A–C**).

The nanobody surface colored according (i) to the hydrophobicity score and (ii) to the electrostatic properties confirmed the divergences in surface electrostatic potentials, especially for the region between CDR1 and CDR3 (**Figure 2D**).

### Subcloning and Purification of BotI-Like-Specific Nbs

The VHH genes were subcloned into the pHEN6 expression vector. The two soluble Nbs were extracted from the bacterial periplasm and purified by Ni-NTA affinity chromatography. Western blotting confirmed the presence of band of 14 kDa (**Supplementary Figure 4**). The average yield of purified NbBotI-01 and NbBotI-17 is estimated at 7.67 and 3.78 mg/L culture, respectively (**Table 1**).

**Table 1:** The kinetic constants and production yield of purified Nbs.

Nanobody	k_on_(M^-1^s^-1^)	k_off_ (s^-1^)	K_D_ (M)	Production yield (mg/L)
NbBotI-01	4.87 ± 0.08×10^6^	4.64 ± 0.15×10^-4^	9.53 ± 0.35×10^-11^	7.67
NbBotI-17	6.52 ± 0.29×10^6^	3.08 ± 0.16×10^-2^	4.72 ± 0.32×10^-9^	3.78

The production yield in milligrams of purified manobody is given per liter of culture. The association and dissociation rate constants were measured by surface plasmon resonance and used to calculate the equilibrium dissociation constant (K_D_). +/− Values present the standard error of the kinetic parameters.

### Affinity Measurements of BotI*-*Like Toxin Binders

**Figure 3:**
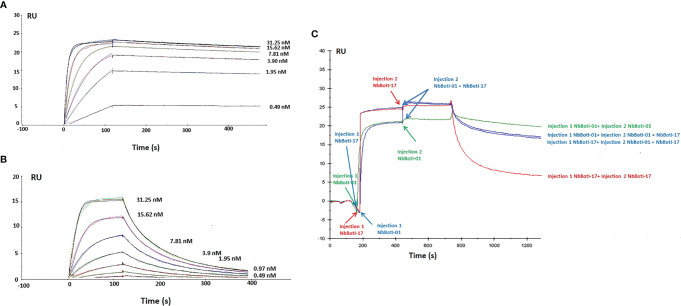
Surface plasmon resonance sensorgrams. Different concentrations from 31.25 to 0.49 nM of NbBotI-01 with six different curves **(A)** and NbBotI-17 with seven different curves **(B)** are flown over the toxin immobilized on the chip. Fittings were with 1:1 stoichiometry and with a 1:1 stoichiometry with RI^2^ and drift. The latter gave consistently better chi^2^ values and was retained. **(C)** Epitope mapping whereby an excess of the monoclonal NbBotI-01 is first injected for 300 s to saturate its epitopes, followed by a mixture of NbBotI-01 with NbBotI-17 for another 300 s. The same experiment was repeated by first injecting an excess of the monoclonal NbBotI-17 followed by a mixture of NbBotI-01 with NbBotI-17 (blue curves). An injection of saturating concentrations of NbBotI-01 (green curve) or NbBotI-17 (red curve) followed by the same concentrations of each Nb, respectively, was measured as a reference.

For both Nbs, two-fold dilution ranging from 125 to 0.49 nM was measured with concentration 31.25 nM in duplicate to confirm reproducibility and two HBS buffer as negative control. In total, nine different concentrations were performed. **Figures 3A, B** show only the curves from the lower concentrations Nb used for the fitting leading to the values displayed in **Table 1**. Thus, curves for 62.5 and 125 nM have been removed. In addition, the curve for 0.98 nM Nb has been removed for NbBotI-01 (due to an air bubble disturbance), which makes a total of six different curves (with a seventh duplicate curve for 31.25 nM, which is hardly noticeable) (**Figure 3A**). **Figure 3B** displays seven curves (eight as there are two curves for 31.25 nM, which nicely superimpose and hardly distinguishable). The kinetic rate constants, *k_on_* and *k_off_* and the equilibrium dissociation constants K_D_ revealed that NbBotI-01 has the best K_D_ value (95 pM) (**Figure 3A**) when compared with the NbBotI-17 K_D_ value (4.7 nM) (**Figure 3B**) (**Table 1**). This nearly 100-fold difference in K_D_ was mainly due to an approximately 100 times lower *k_off_* value for the NbBotI-01 clone.

### Epitope Mapping

The epitope binning revealed that the two nanobodies bind to an overlapping epitope on BotI-like toxin. Indeed, the R_Max_ value for NbBotI-01 (20 RU) is slightly lower than that for NbBotI-17 (25 RU), which is possibly due to some steric hindrance to the NbBotI-01 epitope for a particular orientation of the toxin on the CM5 chip. This can be explained, for example, by assuming that one out of fivve possible chemical linkages of the toxin to the CM5 chip prevented the NbBotI-01 from binding to its epitope, whereas the epitope for NbBotI-17 was always fully accessible independent of the reactive group used during the conjugation of the toxin to the sensor. Consequently, flowing an excess of the NbBotI-01 and NbBotI-17 mixture over the toxin that was first saturated with NbBotI-17 will not lead to an increase in signal. An increase by 4–5 RU might be seen when the epitopes were first saturated with NbBotI-01, as some residual binding sites for the second Nb (NbBotI-17) remained still available after saturating all NbBotI-01 accessible sites. In addition, it seemed that the dissociation kinetics when the mixture of NbBotI-01 and NbBotI-17 passed over the chip (blue curve) are somewhat intermediate compared to the curves obtained when pure NbBotI-01 or pure NbBotI-17 was used (**Figure 3C**). This is exactly what is expected when two Nbs with overlapping epitopes are flown over the immobilised toxin: a fraction of the first Nb will be replaced by the second Nb so as to install a new equilibrium.

### Neutralizing Capacity of Anti-BotI-Like Nanobodies Tested by i.c.v Injection

#### Neutralization of BotI-Like Toxin by the Two Selected Nbs

**Figure 4:**
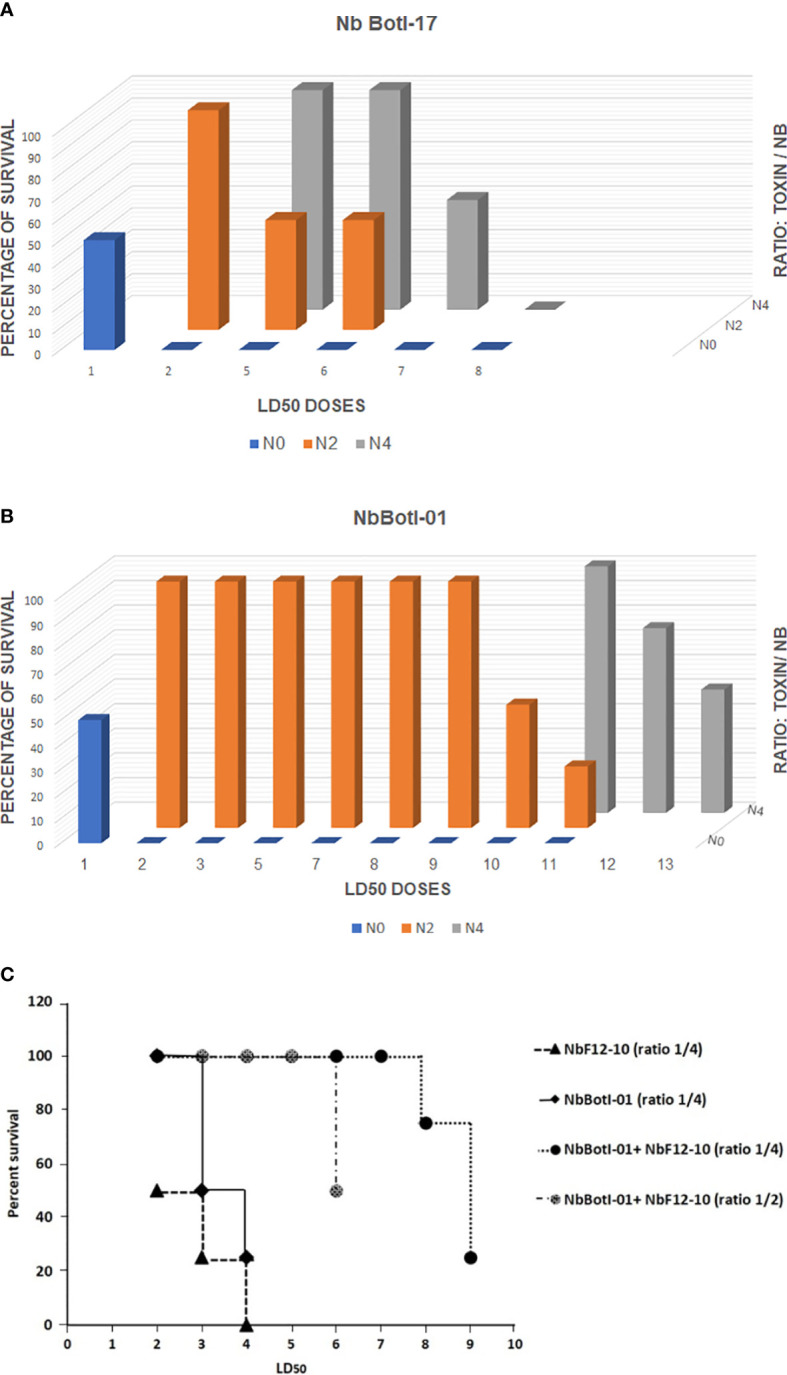
*In vivo* neutralizing capacity of NbBotI-17 **(A)** and NbBotI-01 **(B).** 3D graph bar chart of the neutralization of various LD_50_ doses (X-axes) at various toxin/Nb ratios (Z-axes) (1/0, 1/2, and 1/4 toxin to Nb) and percentage of survival mice (Y-axes). The Nb neutralizing capacity was assessed after preincubation the BotI-like toxic doses and Nbs before administration to mice by i.c.v. injection. The LD_50_ of pure toxin was determined at 40 ng/mouse. Both Nbs display a high neutralizing capacity with NbBotI-01 as strongest neutralizing nanobody. *In vivo* neutralizing capacity of NbBotI-01 and/or bispecific NbF12-10 preincubated with BotG50 **(C)**. NbBotI-01 and bispecific NbF12-10 were mixed with the BotG50 toxic dose and injected into Swiss mice by i.c.v route. The equimolar mixtures of NbBotI-01 and a bispecific NbF12-10 were at a two- or fourfold molar excesses to the BotG50 toxic fraction.

Our data demonstrated that NbBotI-17 at 2 or 4 molar excess neutralized 50% of the toxicity of 6 and 7 LD_50_ of toxin, respectively (**Figure 4A**). Interestingly, under similar conditions, the NbBotI-01, which had the slowest dissociation rate constant, neutralized even higher toxic doses of the toxin. Indeed, for a toxin/Nb ratio of 1/4, approximately 3,520 ng of NbBotI-01 neutralized 100% the toxicity of 11 LD_50_ (440 ng) of toxin, and 4,160 ng of this Nb neutralized in 50% of the mice the toxicity of 13 LD_50_ (520 ng) of toxin (**Figure 4B**).

#### Neutralization of BotG50 Toxic Fraction by NbBotI-01 and by an Equimolar Mixture of NbBotI-01 With a Bispecific NbF12-10

Because of the higher neutralizing capacity of NbBotI-01, we further investigated the neutralization of crude BotG50 and AahG50 toxic fractions. The heterogeneous BotG50 and AahG50 fractions contain different types of toxins (e.g., Na^+^, K^+^, Clˉ channel blockers), which makes it difficult to measure the exact concentration of protein within these fractions. We considered that an OD at 280 nm of 1 corresponded to 0.54 mg/ml (the same extinction coefficient as for the pure toxins). Taking into account that the LD_50_ of BotG50 injected *via* i.c.v. route is 82 ng, we mixed NbBotI-01 at a fixed fourfold molar excess with increasing amounts of BotG50, preincubated the mixture at 37°C, and evaluated the neutralizing activity. We noted that 4/4 and 2/4 mice survived, when injected with 2 LD_50_ and 3 LD_50_, respectively.

We also evaluated the neutralizing capacity of a fourfold excess of the previously developed AahI/AahII bispecific NbF12-10 construct mixed with 2 LD_50_ (~164 ng) or 3 LD_50_ (~246 ng) of BotG50 and showed that only 2/4 and 1/4 mice survived, respectively (**Figure 4C**). We also investigated whether the lethal effect of the BotG50 toxic fraction could be further neutralized by using an equimolar mixture of NbBotI-01 with AahI/AahII-bispecific NbF12-10 (37). Interestingly, at a fourfold excess, this mixture neutralized 100% of the highly lethal effect of 7 LD_50_ of BotG50. Moreover, with 6 LD_50_ of BotG50 toxic fraction, only 50% of the mice died when the ratio of the mixture over BotG50 was reduced from 4:1 to 2:1 (**Figure 4C**).

#### Neutralization of AahG50 Toxic Fraction by NbBotI-01 and an Equimolar Mixture of NbBotI-01 With a Bispecific NbF12-10

Additionally, we evaluated the neutralizing activity of the NbBotI-01 at a fixed fourfold molar excess to increased amount of AahG50 (LD_50_ = 52 ng) preincubated at 37°C. We noticed that no mice survived when injected with 2 LD_50_ of AahG50, demonstrating that BotI-related toxins are not significantly represented in the AahG50 fraction.

In addition, 4/4 mice survived when injected with fourfold molar excess of NbF12-10 preincubated with 11 LD_50_ of AahG50, and only 2/4 survived when injected with 13 LD_50_. As expected, fourfold molar excess of the mixture (NbF12-10 and NbBotI-01) showed a similar neutralizing capacity and ensured the survival of all mice (4/4) injected with 11 LD_50_, whereas with 13 LD_50_ of AahG50, 50% of mice (2/4) survived.

## Discussion

In the Maghreb region, many countries encounter scorpion envenoming accidents. Some dangerous species of scorpion belong to the family of Butidae, and their venom consists of a complicated mixture, mainly of protein and peptides with neurotoxic effects.

Despite therapeutic improvement against various diseases, up to now, there are no effective vaccines against scorpion or snake envenoming. As a result, antivenom immunotherapy continues to be the mainstay of treatment for scorpion sting victims. Nanobodies, the single domain, antigen-binding variable fragments (VHH) derived from heavy-chain only antibodies (HCAbs) naturally occurring in camelid serum represent a very promising approach for generating unique anti-venoms to treat severe scorpion envenoming cases (25). The VHH exhibits the specificity of a monoclonal antibody, while it is much smaller in molecular weight. Consequently, the size of the nanobody matches closely the size of the scorpion toxins, allowing a nearly similar penetration and distribution in envenomed tissues.

In our previous work, we have selected Nanobodies directed against Aah*I* and Aah*II* toxins, the two most poisonous polypeptides from *A. australis hector* scorpion venom that belong to the first and second structural and antigenic homology groups, respectively. We successfully identified multiple Nbs of sub-nanomolar affinity against the Aah*II* toxin (35), and interestingly, NbAahII10 exhibited also an excellent neutralization capacity. This outstanding candidate has also been the subject of a humanization effort by site-directed mutagenesis (36). Furthermore, a panel of 13 Nbs against Aah*I* toxin were identified, of which NbAahIF12 showed the highest affinity (K_D_ of 100 pM). We then investigated the generation of a bispecific nanobody, NbF12-10, with the aim of developing a more efficient antivenom therapy against *Androctonus* scorpion envenoming (37). This bispecific format with a MW of 29 kDa is able to recognize both groups of active toxins in the sub-nanomolar range. It was also proven to be more protecting than the classical polyclonal antiserum tested into envenomed Swiss mice (37).

The current essential challenge is to overcome the structural and antigenic diversity of the toxins within the venom of various scorpion species that occur in the same region, which may limit the antivenom efficacy, by developing nanobodies that specifically recognize and neutralize BotI-related toxins. We first purified and identified the most toxic molecules analogous to BotI toxin. The most toxic molecule that is recognized by BotI-specific rabbit polyclonal antibodies shared a high sequence identity to the primary sequence of the BotI toxin, exceeding 90%. In particular, the sequence displayed the conserved 8-QPE-10 exposed loop, a characteristic of alpha-toxins (24). Four structurally divergent residues located at four distinct positions (I5K, Q18K, S20N, and L25C) have not been reported previously. Indeed, the presence of two successive cysteines at positions 25–26 confirm that the novel sequence, homologous to BotI, might display slight differences in 3D topology with the highly antigenic toxins belonging to the BotI group. The LD_50_ of the purified molecule referred to as BotI*-*like toxin is 40 ng/mouse.

This BotI-like toxin was further used to immunize a dromedary. On the basis of ELISA results, the immunization of the dromedary with the purified BotI-like toxin stimulated the production of a high titer of polyclonal antibodies. Therefore, the BotI-like toxin molecule is immunogenic and elicited a humoral response. These results are in line with our previous studies using scorpion toxin (36, 37), metallo-beta-lactamase enzyme (55), or Tenacin protein (56) to raise specific immune responses in dromedary. We then constructed an immune VHH library of 3.0×10^6^ individual clones, 87% of which carried a phagemid with an insert of the correct size of a VHH. The results of polyclonal phage ELISA on phage eluted from each round of biopanning confirmed the enrichment of antigen-specific phages during the consecutive rounds of panning. The *in vitro* selection of recombinant binders from this BotI-specific VHH library retrieved two distinct binders according to their CDR3 sequence, referred to as NbBotI-01 and NbBotI-17. An explanation for this limited sequence diversity of binders might be linked to the size of the library (3 × 10^6^ cfu/ml) or a limited antigenicity of the toxin whereby only the highest affinity binders dominated the competition for the single or limited number of epitopes. Translated amino acids obtained from DNA sequencing revealed that one of the two VHH clones, NbBotI-17, possessed all hallmark residues corresponding to a VHH fragment (32), whereas the other binder NbBotI-01 is a conventional-like VHH, since the first amino acid position of the framework region-4 deviated from the conserved Trp in the consensus VH anchoring site for the VL by its substitution into an Arg. Remarkably, 3D models of NbBotI-01 revealed a clustering of positively charged area at the CDR3 caused by three Arg at position 109, 114, and 116 and one Arg118 at the start of framework region-4. Possibly, the Arg116 and Arg118 of NbBotI-17 CDR3 and framework region-4 might be involved in the BotI epitope recognition, which may explain the increased neutralizing capacity, as previously reported (49).

Furthermore, the electrostatic surface confirmed the divergences in surface electrostatic potentials, especially for the region between CDR1 and CDR3.

The VHH genes were subcloned into a pHEN6 expression vector and transformed into *E. coli*. The proteins from the *E. coli* periplasm were extracted, and the nanobodies were purified to homogeneity by IMAC and gel filtration as demonstrated by Western blot analysis. The production yield of purified NbBotI-01 and NbBotI-17 is estimated at 7.67 and 3.78 mg/L, respectively. Expression of the nanobodies into the bacterial cytoplasm or switching to fermentation of nanobodies in yeasts might increase the production level (57).

The affinity constant of the two selected NbBotI-17 and NbBotI-01 was in single-digit nanomolar and low picomolar range, respectively. This is completely in line with previous higher affinities reported for nanobodies from immune libraries directed against various antigens (36, 37, 58–61). These binding properties might be critical in obtaining the best possible toxin neutralization *in vivo*, although the actual epitope that is recognized will also be essential. However, epitope binding revealed that the two Nbs do not bind to exactly the same epitope, but there is definitely an overlap between their epitopes.

Finally, the neutralizing capacity was assessed. We employed a standard 4 and 2 molar excess of Nb over toxin, which is in line with the conditions used in neutralization assays with other Nb constructs (36, 37, 39). Our data demonstrated that the preincubated mixture of one-fourth of toxin to NbBotI-17 stoichiometry administrated by i.v.c route fully neutralized the BotI-like lethal effect in mice. Remarkably, NbBot-01, with the slowest dissociation rate constant, neutralized even higher toxic doses of the BotI-like toxin. Indeed, this nanobody neutralized 100% of the toxicity of 11 LD_50_ of BotI-like toxin when NbBotI-01 was present at a 4 molar excess.

Furthermore, we investigated the neutralization of the lethal effect of the more complex BotG50 toxic fraction by using an equimolar mixture of NbBotI-01 with a bispecific NbF12-10 previously constructed and directed against *AahI* and *AahII* toxins from *A. australis hector* scorpion venom (37). Interestingly, the mixture was able to neutralize 100% the highly lethal effect of 7 LD_50_ of BotG50, while NbBotI-01 or NbF12-10 alone were neutralizing only 50% of the toxicity effect of 3 and 2 LD_50_ of BotG50, respectively.

As expected, the mixture ensured the survival of all mice (4/4) injected with bispecific NbF12-10 and 11 LD_50_ of AahG50, whereas with 13 LD_50_, 50% of mice (2/4) survived. We noticed that the addition of a 4 molar excess of NbBotI-01 to the 4 M of NbF12-10 preincubated with 13 LD50 of AahG50 did not further increase the neutralizing capacity of the mixture compared to the injected NbF12-10. This result was explained by the absence of toxin belonging to the third valance within Aah venom.

## Conclusion

In this study, we reported the successful selection of two Nbs with high neutralizing potency against BotI-like toxin purified from *B. occitanus tunetanus* scorpion venom. NbBotI-01 had the highest affinity for BotI-like toxin and was shown to be the most potent BotI-like toxin neutralizer. Furthermore, administration of an equimolar mixture of NbBotI-01 with the Aah toxin-specific NbF12-10 neutralizer to scorpion-stung victims might be a good antivenom therapeutic treatment for the MENA region where both scorpions live. These candidates will be either engineered and/or matured in trimeric Nb formats for better synergic effects. Although recent reports indicate that Nb-derived material is well tolerated in human volunteers without any serious adverse effects (http://www.ablynx.com), we propose to humanize these trimeric formats using the framework of the universal humanized hNbBCII10FGLA followed by investigating their pharmacokinetic and more precisely the plasmatic half-life for antivenom immunotherapeutic potential use.

## Data Availability Statement

The original contributions presented in the study are included in the article/**Supplementary Material**. Further inquiries can be directed to the corresponding authors.

## Ethics Statement

The animal study was reviewed and approved by the Ethics Committee of Pasteur Institute of Tunis.

## Author Contributions

RBA designed and performed the experiments related to the library construction, VHH selection, and characterization of nanobodies. She also analyzed the data and wrote the manuscript. AK helped in molecular structure investigations. FI helped in library construction. SD contributed to nanobody selection. EH helped in ELISA experiments. CV contributed by SRP measurements. AF helped in mice experiments. ZB helped in Animal Ethic protocols and contributed to the dromedary veterinary management and immunizations. HM contributed by toxin sequencing. MEA contributed by funding. SM helped in data analysis and manuscript editing. BB-Z helped in data analysis and contributed by funding, manuscript writing, and editing. All authors contributed to the article and approved the submitted version.

## Funding

This work was partially supported by Pasteur Institute, Ministry of Health &Ministry of High Education and Scientific Research of Tunisia. Part of the grant was supported by the VenoMATIcs project funded by the regional International Network of Institute Pasteur (IPIN MATI region).

## Conflict of Interest

The authors declare that the research was conducted in the absence of any commercial or financial relationships that could be construed as a potential conflict of interest.

## Publisher’s Note

All claims expressed in this article are solely those of the authors and do not necessarily represent those of their affiliated organizations, or those of the publisher, the editors and the reviewers. Any product that may be evaluated in this article, or claim that may be made by its manufacturer, is not guaranteed or endorsed by the publisher.

## Footnotes

1
https://www.rosettacommons.org/software/license-and-download

## Supplementary Material

Supplementary Figure 1 BotI like toxin-specific immune response elicited in dromedary. ELISA was performed to evaluate the immune response of Camelus dromedary using immune serum collected at the end of immunization program. Preimmune serum was used as a negative control Error bars represents standard deviation.

Supplementary Figure 2 Analysis of two PCR products on gel agarose. **(A)** The first PCR presents two bands of 600–700 and 900 bp corresponding to VHH-Hinge-CH2 and VH-CH1-CH2 precursors of heavy chain and conventional antibodies, respectively. **(B)** The second PCR product harbors VHH fragments with 400 bp. M, molecular weight marker.

Supplementary Figure 3 Assessment of biopanning steps with phage ELISA. 10^11^ eluted phages from each round of panning were tested for binding activity against toxin (1µg/ml) in comparison to negative control (BSA). The antigen-specific bound phages were detected with HRP-conjugated anti-M13 monoclonal antibody. The second and the third rounds of panning showed the highest immune reactivity.

Supplementary Figure 4 Western blotting under reducing conditions. NbBotI-17 (line 1) and NbBotI-01 (line 2) were detected with anti-His antibody-PO.
