# Supplementary material for: Neutralizing Dromedary-Derived Nanobodies Against BotI-Like Toxin From the Most Hazardous Scorpion Venom in the Middle East and North Africa Region
Source: Front Immunol. 2022 Apr 19;13:863012. doi: 10.3389/fimmu.2022.863012 (PMC9063451; doi:10.3389/fimmu.2022.863012)
Supplement: Supplementary Figure 1 — BotI like toxin-specific immune response elicited in dromedary. ELISA was performed to evaluate the immune response of Camelus dromedary using immune serum collected at the end of immunization program. Preimmune serum was used as a negative control Error bars represents standard deviation. [file DataSheet_1.docx]

**Supplementary figure S1**
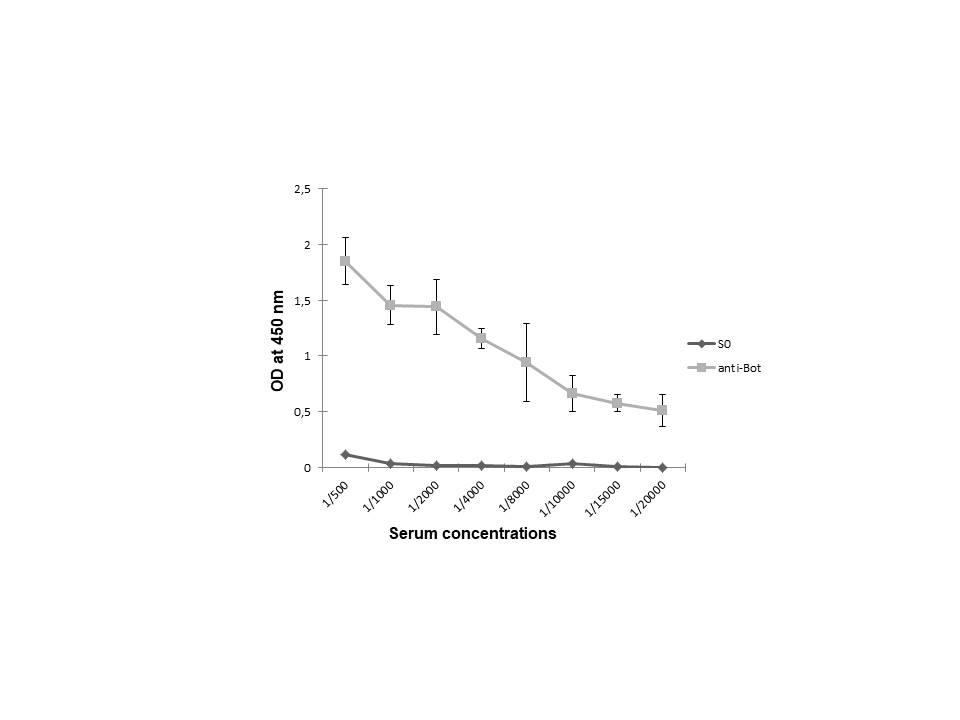


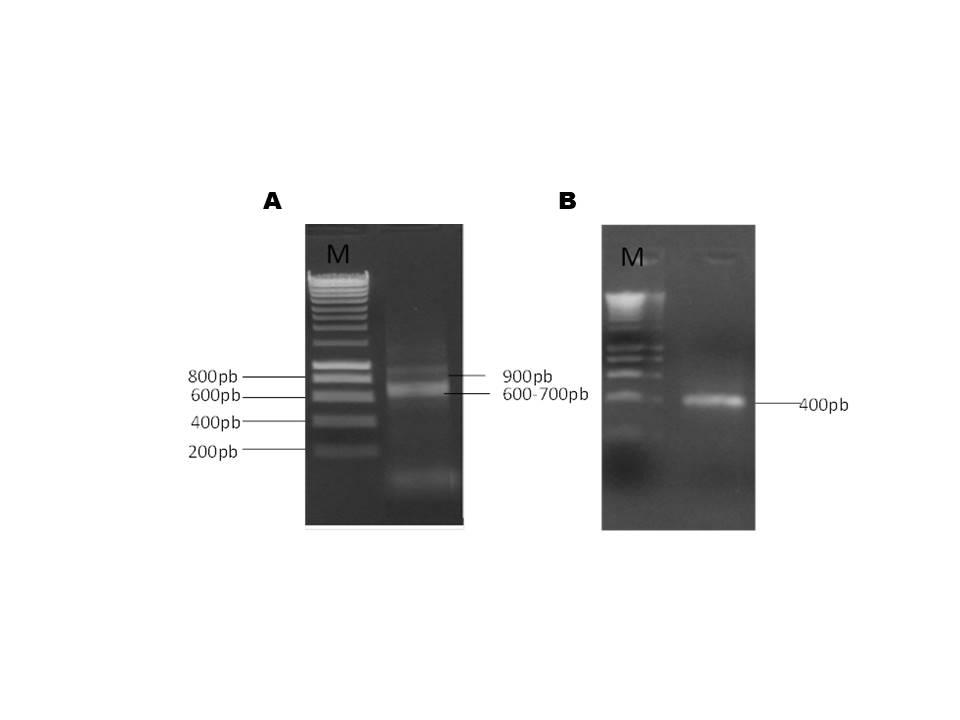
**Supplementary figure S2**


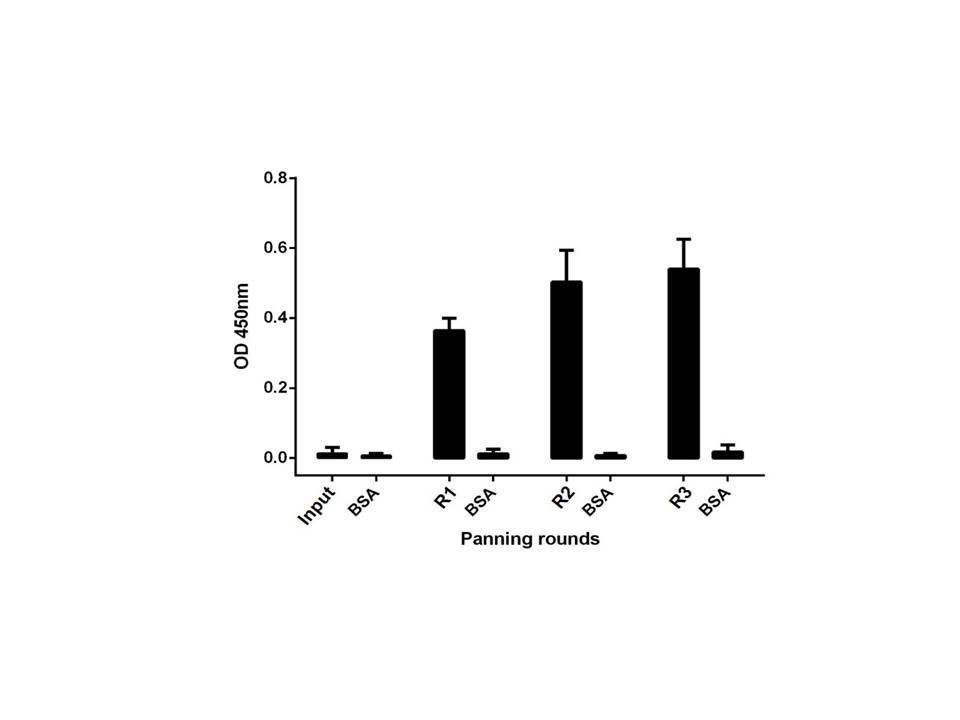


**Supplementary figure S3**


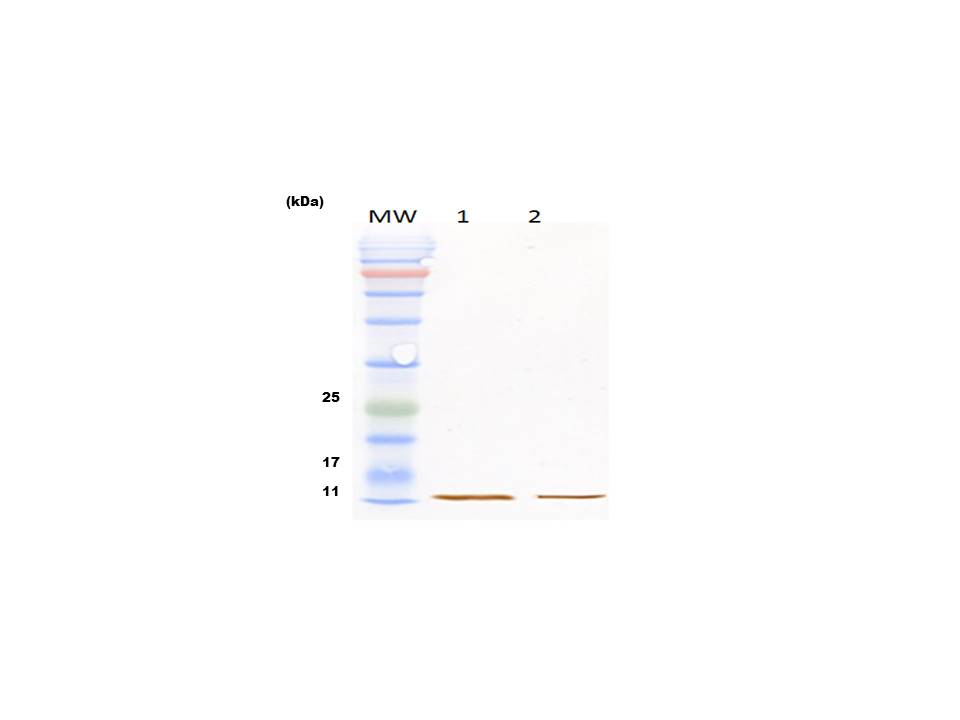


**Supplementary figure S4**
